# Coupled partitioning of Au and As into pyrite controls formation of giant Au deposits

**DOI:** 10.1126/sciadv.aav5891

**Published:** 2019-05-01

**Authors:** C. Kusebauch, S. A. Gleeson, M. Oelze

**Affiliations:** 1 GFZ German Research Centre for Geosciences, Telegrafenberg, 14473 Potsdam, Germany.; 2 Freie Universität Berlin, Institute for Geological Sciences, Malteserstraße 74-100, 12249 Berlin, Germany.

## Abstract

The giant Carlin-type Au deposits (Nevada, USA) contain gold hosted in arsenic-rich iron sulfide (pyrite), but the processes controlling the sequestration of Au in these hydrothermal systems are poorly understood. Here, we present an experimental study investigating the distribution of Au and As between hydrothermal fluid and pyrite under conditions similar to those found in Carlin-type Au deposits. We find that Au from the fluid strongly partitions into a newly formed pyrite depending on the As concentration and that the coupled partitioning behavior of these two trace elements is key for Au precipitation. On the basis of our experimentally derived partition coefficients, we developed a mass balance model that shows that simple partitioning (and the underlying process of adsorption) is the major depositional process in these systems. Our findings help to explain why pyrite in Carlin-type gold deposits can scavenge Au from hydrothermal fluids so efficiently to form giant deposits.

## Teaser

The precipitation of As-rich pyrite (“fool’s gold”) can scavenge gold from hydrothermal fluids to form giant ore deposits.

## INTRODUCTION

The average concentration of Au in the continental crust is 2.5 parts per billion (*1*) and is orders of magnitude too low to be mined. To form an economic gold deposit, Au must be extracted from a source, transported in hydrothermal solutions, and then precipitated in a very efficient way in a small area accessible for mining. The Carlin-type gold deposits (CTGDs) of Nevada (USA) contain the second largest accumulation of Au on Earth and currently account for ~5% of total world Au production (*2*–*4*). The ultimate source of the Au in these deposits is debated, with some workers arguing for a magmatic-hydrothermal origin of Au (*3*, *4*) and others suggesting that Au was remobilized from pre-enriched (meta)sediments (*5*–*7*). Irrespective of the source, the hydrothermal fluids that transport the Au are well characterized [*T*, 180° to 240°C; pH 5; >0.01 *m* H_2_S; 1 to 4 mole percent (mol %) CO_2_; 3 to 6 weight % (wt %) NaCl] (*2*, *8*, *9*), and both models require an effective depositional mechanism to form the giant Au deposits. The gold in CTGDs is almost exclusively hosted in arsenian pyrite either as nanometer-sized particles of native Au^0^ or, more commonly, as dissolved Au^+1^ in the pyrite structure depending on its As content (*10*–*12*).

Gold-rich pyrite forms via the interaction of an Au- and H_2_S-containing fluid with reactive iron in Fe-bearing carbonates of the wall rock (*13*, *14*) or a Fe-bearing fluid (*15*, *16*). The resulting H_2_S consumption leads to the destabilization of dissolved Au-HS complexes and, consequently, to the precipitation of Au (*7*, *13*, *14*). In addition to this desulfidation of the fluid (or sulfidation of the wall rock), earlier workers suggested that chemisorption of Au onto an As-bearing, but Fe-deficient, pyrite surface could be an effective process to enrich Au in hydrothermal pyrite without reducing H_2_S concentration (*17*, *18*). This could explain why Au deposits with high As (>1 wt %) in pyrite (i.e., CTGDs and epithermal) characteristically have higher Au contents than deposit types with low As pyrite (i.e., orogenic and porphyry), although the Au concentrations of the Au-transporting fluid are similar or even higher (*10*, *19*). Up to now, no data have been published that quantify the amount of Au deposited by either of the two processes (i.e., sulfidation and chemisorption).

The aim of this study was to experimentally determine the partitioning of Au between hydrothermal fluids and the newly formed As-bearing pyrite under conditions similar to CTGD formation. Our experimental results show that Au strongly partitions into newly formed pyrite depending on its As concentration. Furthermore, our findings allow a quantification of Au partition coefficients, which, in turn, can be used to explore the dominant processes controlling the formation of CTGDs.

## RESULTS

### Coupled As and Au partitioning during experimental siderite replacement by pyrite

**Fig. 1:**
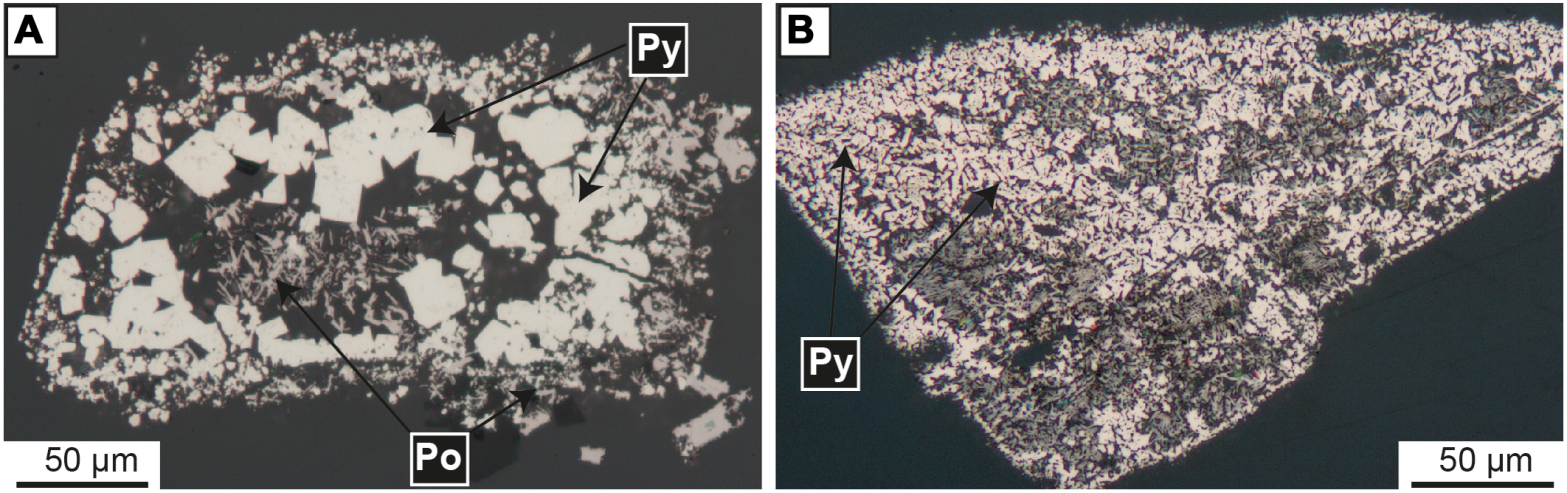
Pyrite formed by experimental replacement of siderite (FeCO_3_). In experiments (**A**) Sd2Py26 and (**B**) Sd2Py52. Euhedral to subhedral clusters of As- and Au-bearing pyrite (FeS_2_) are formed when fluid (rich in H_2_S)–mediated dissolution-reprecipitation of siderite occurs. Minor amounts of pyrrhotite (Po) needles formed interstitially between pyrite (Py) clusters, either in equilibrium with pyrite or as a late phase during cooling of the experiments.

**Fig. 2:**
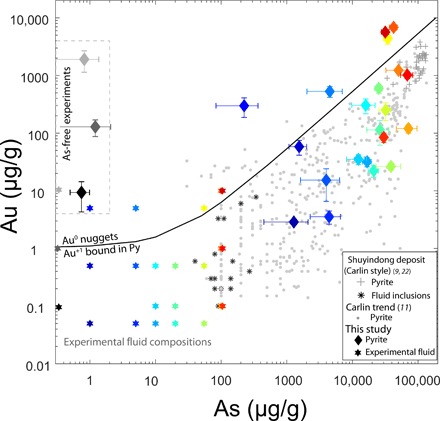
Compositions of experimental fluid and coexisting hydrothermal pyrite. Experimental products are coded by the same color. The black line is the empirically defined upper limit of Au^+1^ in pyrite as a function of As concentration of the pyrite (*10*, *11*), pyrite falling above the limit contains Au as (nano)nuggets, and samples that plot below characteristically have Au dissolved as Au^+1^ in the pyrite structure (*12*). Natural pyrite compositions for CTGD from Nevada (•) (*11*) and Shuyindong (+) (*22*) are plotted together with limited published data for fluid inclusions (*) (*9*). Experimental fluids: Without As (gray), low As (bluish colors), and high As (reddish colors) produce pyrite that has As and Au concentrations that are orders of magnitude higher than the fluids and agrees well with natural pyrite.

**Fig. 3:**
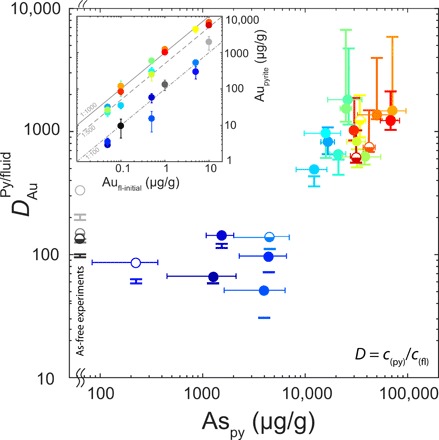
Gold partition coefficients (*D* values) between pyrite and fluid as a function of the As concentration in pyrite. Inset: Concentration of Au in the newly formed pyrite compared to the initial Au composition of the experimental fluid; color coding identical to Fig. 2. Circles represent *D*_opt_ values, error bars for *D* values represent *D*_min_ and *D*_max_ (see Materials and Methods), and error bars for As is the SD of LA-ICPMS measurements. Filled symbols represent pyrite composition falling below the Au^+1^ solubility limit for arsenian pyrite (*11*). Half-filled symbols represent pyrite above the limit, but LA-ICPMS analysis does not show substantial formation of Au^0^ nuggets. Empty symbols represent pyrite falls above limit, and LA-ICPMS analysis indicates Au^0^ nuggets (see fig. S1)

The partitioning of As and Au into pyrite was studied by replacing siderite (FeCO_3_) by pyrite (FeS_2_) by reacting the Fe carbonate with H_2_S-containing aqueous fluids under experimental conditions similar to those of CTGD formation (i.e., 200°C, fluid-dominated conditions, 0.05 *m* H_2_S, and slightly acidic; see Materials and Methods for details). The newly formed pyrite occurs either as 10- to 40-μm–sized euhedral crystals or as cluster of smaller subhedral grains (Fig. 1). To cover a broad range of potential CTGD fluid compositions (*4*, *9*, *20*), the concentration of As and Au in the experimental fluid was varied between 0 to 100 μg/g and 0.05 to 10 μg/g, respectively (stars in Fig. 2). The concentrations of As and Au in the newly formed pyrite were quantified by laser ablation inductively coupled plasma mass spectrometry (LA-ICPMS) (see Materials and Methods) and are orders of magnitude higher than in the fluid (Fig. 2 and table S2). Nernst partition coefficients [*D* = *c*_(py)_/*c*_(fl)_, where *D* is the Nernst partition coefficients and *c* is the concentration of As or Au in pyrite and fluid, respectively] were calculated in three different ways (see Materials and Methods) to account for compositional variations within individual experimental runs. Best-fit *D* values (*D*_opt_) vary as a function of As concentration of the fluid between 330 and 2660 for As (*21*) and between 50 and 1800 for Au, respectively (Fig. 3 and table S2). For As, the lowest *D* values correspond to the highest As fluid concentration (*21*). Contrastingly, the *D* values of Au increase with As concentration of the pyrite and reach values of ~10^3^ at 3 to 7 wt % As in pyrite; these compositions are also characteristic for CTGDs (Fig. 2).

## DISCUSSION

### Au in natural CTGD hydrothermal fluids

The observed partition coefficients of our experiments are representative for natural ore systems such as CTGDs. In CTGDs, the lack of large fluid inclusions, the high detection limits, and the low concentrations of trace elements in individual fluid inclusions mean that fluid trace element data are rare and only a few studies that published Au and As values for coexisting fluid inclusions and pyrite exist (*4*, *9*, *22*, *23*). In most cases, Au concentrations of fluid inclusions are below the analytical detection limit (~1 to 5 μg/g depending on the salinity of the inclusion), and only exceptionally large ore fluid inclusions from Carlin style deposits Shuiyindong and Yata (*9*, *22*, *23*) yielded reliable Au concentrations between 0.3 and 8 μg/g (Fig. 2). Gold concentrations of coexisting arsenian pyrite from these deposits are three orders of magnitude higher, confirming our experimentally derived partition coefficients (Fig. 3). Using the newly constrained *D* values (1100 on average for arsenian pyrite) and published ore stage pyrite LA-ICPMS and secondary ion mass spectrometry data (*10*, *11*, *15*, *24*), we calculate Au concentrations in the Nevada CTGD ore fluids ranging from below 0.1 to ~2.3 μg/g. These concentrations are comparable with those suggested for a magmatic-hydrothermal origin of CTGD fluids (*3*, *4*, *19*). A magmatic-hydrothermal fluid is capable of transporting Au (10 μg/g) as Au(HS)S_3_^−^ at a total dissolved S level of 2 wt % and a temperature of ~600°C (*19*). The reduction in S concentrations (at *T* > 400°C) to levels characteristic for CTDGs (i.e., 0.01 to 0.1 *m* H_2_S corresponding to 0.032 to 0.32 wt % S) will decrease the maximum dissolved Au concentrations to values between 0.1 and 0.7 μg/g as the S_3_^−^ complexes are destabilized. Because of the retrograde stability of the Au(HS)_2_^−^ complex (*25*, *26*), further cooling of the already Au-depleted fluid from magmatic-hydrothermal (~400°C) to CTGD (~200°C) temperatures will not lead to supersaturation of Au. Only the interaction of the fluid with reactive iron from the carbonaceous CTGD host rock and the precipitation of arsenian pyrite will lead to the deposition of Au.

Although As and Au concentrations of natural ore stage pyrite can vary over orders of magnitude within deposits or even within single grains (Fig. 2), the As/Au ratio in cogenetic pyrite is found to be remarkably constant (*10*, *11*, *27*, *28*). In light of our newly constrained partition coefficients, this is not surprising, as *D* values for As and Au are in the same order of magnitude (i.e., 10^2^ to 10^3^). Consequently, the compositional changes observed in the ore stage pyrite for one element, which indicates an evolution of the fluid composition, will coincide with changes of the other element. Hence, As/Au ratios of the pyrite are representative of As/Au ratios of the fluid. The constant As/Au ratios cannot be explained by a desulfidation of the fluid, as As and Au are speciated by different complexes in the fluid. Destruction of Au(HS)_2_^−^ complexes due to pyrite formation would lead to a supersaturation of Au in the fluid, but the major uncharged As(OH)_3_^0^ species (*29*, *30*) will not be affected by this process. Hence, if desulfidation of the ore fluid is the major ore-forming process, then the As/Au ratios in cogenetic pyrite should vary over orders of magnitude. As this is not observed, we propose Au scavenging by partitioning (= sorption + incorporation during growth) during pyritization of the host rock to be the major ore-forming process in CTGDs.

### Desulfidation of fluid versus Au scavenging by partitioning

To assess the role that scavenging of Au by partitioning into pyrite plays during formation of CTGDs, we applied a mass balance model together with thermodynamic modeling to calculate the Au deposition during fluid-rock interaction (see Materials and Methods and the Supplementary Materials for details). To quantify the importance of the two Au depositing processes during ore formation, we distinguished between “sulfidation” of the rock (or equivalent “desulfidation” of the fluid) and pyritization. We define sulfidation as the process whereby pyrite is formed by the reaction of reactive iron from the host rock with H_2_S from the fluid. This leads to the destabilization of Au-HS complexes and, therefore, to supersaturation of Au in the fluid. In contrast, we use the term “pyritization” to refer to the precipitation of pyrite due to fluid rock interaction (similar to the first part of sulfidation), which does not involve the destruction of Au-HS complexes. During pyritization, Au will be scavenged from the fluid purely as the result of sorption onto and incorporation into the growing surface of the (arsenian) pyrite and is, therefore, an expression of partitioning between fluid and pyrite.

To model the sulfidation, we calculate the solubility of Au as a function of H_2_S using the thermodynamic software package PHREEQC (*31*), applying its implemented “llnl” database with added equilibrium constants for the Au-HS-Cl complexes (table S3). The solubility of Au at H_2_S concentrations of 0.01 to 0.1 mol/kg ranges between 4.2 and 185 μg/g, respectively, and is one to two orders of magnitude higher than the calculated and measured Au concentrations of CTGD fluids (see above). Therefore, it is reasonable to assume that Au-transporting CTGD fluids are largely undersaturated in Au with respect to native Au (*3*, *12*, *32*). Thus, Au deposition caused by sulfidation will only start when the fluid becomes supersaturated with respect to Au. Pyrite that is formed before reaching Au supersaturation will sequester Au because of partitioning.

**Fig. 4:**
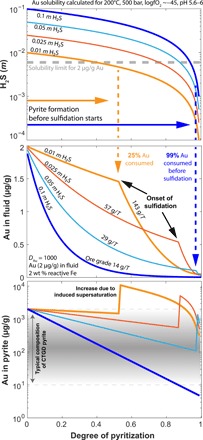
Modeled evolution of H_2_S and Au in hydrothermal fluids and pyrite for CTGD systems. Reaction of hydrothermal fluids with reactive Fe at concentrations common in CTGD leading to Au-rich pyrite. Numerical model assumes complete consumption of H_2_S from the fluid to form pyrite. Au concentration depends either on partitioning (at low degrees of pyritization) or supersaturation due to sulfidation (at high degrees of pyritization). The gold solubility limit will decrease during pyritization as Au is consumed because of partitioning. The onset of sulfidation causes a marked increase in modeled Au concentration in pyrite. Under these conditions, typical compositions of CTGD pyrite are only formed at the very end of pyritization (gray field). When partitioning is the major ore-forming process, typical CTGD compositions are produced during the course of pyritization. Ore grades are calculated from amounts of fluid and rock and initial Au concentration (see the Supplementary Materials).

To evaluate the amount of Au deposited as a function of partitioning and pyritization, we set up a numerical mass balance model (see Materials and Methods). The degree of pyritization in our model is equal to the amount of H_2_S consumed from the fluid and fixed in the newly formed pyrite. In other words, at low degrees of pyritization, most of the initial H_2_S is still present in the fluid, whereas increasing degrees of pyritization result in lower H_2_S concentrations of the fluid (Fig. 4) down to concentrations of pyrite solubility at a given logfO_2_ of −45 (i.e., <0.0001 *m* H_2_S). Initial conditions for numerical modeling of the hydrothermal fluid represent compositions and conditions of natural ore fluids that formed CTGD (Table 1): 0.01 to 0.1 *m* H_2_S, Au [2 μg/g; corresponding to Au (2000 μg/g) in coexisting arsenian pyrite], and a *D* value of 1000. Reactive Fe in the wall rock is assumed to be sufficiently abundant to completely react all H_2_S until the pyrite saturation is reached, an assumption that is validated by the presence of remnant mineral phases containing reactive Fe that have not reacted or precipitated during cooling of the fluid (*14*, *15*).

**Table 1:** Variables and conditions used in thermodynamic and mass balance models.

**Variable**	**Value**	**Literature source**
H_2_S	0.01–0.1 *m*	(*5*)
Temperature	200°C	(*5*)
NaCl	5 wt %	(*5*) and (*14*)
CO_2_	2 mol %	(*5*)
Reactive Fe in wall rock	0.5–2 wt %	(*13*); given as siderite in thermodydnamic modeling
pH	~5.6	(*5*) and (*14*); buffered by calcite and siderite dissolution
LogfO_2_	~−45	(*5*); buffered by calcite and siderite dissolution
Au in fluid	0.5–2 μg/g	(*9*) and this study
*D*_Au_	100–6000	This study

Figure 4 shows the modeled evolution of the fluid H_2_S concentration during pyritization for different initial H_2_S conditions. Even with the lowest H_2_S concentrations (i.e., 0.01 *m*), the hydrothermal fluid will always be undersaturated in Au at the initial stages and will only become supersaturated when H_2_S intersects the solubility limit, which is the starting point for sulfidation (Fig. 4).

The evolution of fluid Au concentration due to pyritization was modeled with the newly constrained *D* values for Au (Fig. 3) in arsenian pyrite. From this simple model, we calculate how much Au was sequestered before the solubility limit of Au is reached (Fig. 4). It becomes clear that pyritization is the governing process for ore generation at high H_2_S concentrations because a large quantity of pyrite is formed (containing hundreds to thousands of parts per million Au) before sulfidation starts. In low H_2_S systems, sulfidation becomes more important as the solubility limit is reached before much new pyrite is formed. In addition, As plays an important role in this system as the *D* values for Au depend on As in pyrite (Fig. 3). If partition coefficients for Au are low (*D* <100) because of low As concentrations in pyrite, then the pyrite formation is not able to sequester a lot of Au before the onset of sulfidation (fig. S3A). In contrast, if the fluids have lower (~0.5 μg/g) Au concentrations [characteristic for most of CTGD ore stage pyrite with Au (~500 μg/g)] (*10*, *11*, *15*), then the model suggests that the system will be undersaturated in Au throughout the course of pyritization. This, in turn, will shift the onset of sulfidation toward higher degrees of pyritization (fig. S3B), and Au deposition by partitioning will be favored. The opposite effect can be observed if the Au solubility decreases because of changes of fO_2_ or pH of the fluid (fig. S4). Nevertheless, in all our considerations, we assume an almost complete consumption of S from the fluid to reach undersaturation of pyrite; this may be an overestimation, and more reduced S in the form of H_2_S may still be present in the fluid. This, in turn, would lead to changes in the level of Au supersaturation and a greater influence of partitioning over sulfidation.

### Adsorption onto growing pyrite surface

Partitioning in hydrothermal environments is strongly linked to the adsorption of dissolved species onto a growing surface and is most likely the underlying process that controls the enrichment of Au in arsenian pyrite (*17*, *18*, *33*). Maximum adsorptions of As and Au [as As(OH)_3_ and Au^+1^ as Au(HS)^0^] under ambient conditions are ~1.7 wt % (*33*) and 91 to 340 μg/g (*18*) on pure FeS_2_ surfaces. It is reasonable to assume that adsorbed As is completely incorporated into growing pyrite, as on one hand, concentrations in natural pyrite have comparable values to maximum adsorption values (*10*, *11*), and on the other hand, substitution of As for S forms an ideal solid solution at concentrations below 4 mol % at the temperatures of interest (*34*). In the case of Au, there is a discrepancy between the concentrations of adsorbed Au(HS)^0^ onto the surface of pure pyrite (*18*) and dissolved Au^+1^ in natural and synthetic pure pyrite, which is one to two orders of magnitude lower (*11*, *35*). As the incorporation of Au^+1^ into pyrite is linked to the sorption of Au-HS complexes onto a growing pyrite surface, changes in the surface chemistry potentially affect the mechanism of incorporation. In As-free systems, adsorbed Au^+1^ [i.e., as Au(HS)^0^ species] is reduced to Au^0^ and forms (nano)nuggets of native Au as inclusions in pyrite (*18*, *36*), which is also observed in As-free experiments of this study (see figs. S1 and S2). If As^−1^ substitutes on the surface for S^−2^, then the reduction of Au^+1^ to Au^0^ might well be prohibited and Au^+1^ could be directly incorporated together with As^−1^ into the pyrite structure. An As-bearing pyrite surface might also be less negatively charged than a pure pyrite surface, allowing for not only adsorption of uncharged Au(HS)^0^ complexes but also adsorption of the more abundant species Au(HS)_2_^−^. This could explain the higher concentrations of dissolved Au than those predicted by adsorption experiments with As-free pyrite (*18*) and is in agreement with our findings of increasing *D* values of Au with increasing As in pyrite. Once Au-HS complexes [i.e., Au(HS)^0^ and/or Au(HS)_2_^−^] are adsorbed to the surface, Au^+1^ can be easily incorporated into pyrite, as an excess of dissolved Fe^+2^ [e.g., from dissolution of Fe-bearing carbonate (*13*, *14*) or from an additional fluid (*15*, *16*)], on the one hand, will lead to rapid growth of new pyrite and, on the other hand, might use S of the adsorbed Au-HS complexes for formation of additional pyrite. These considerations imply that the occurrence of dissolved Au^+1^ or Au^0^ nanonuggets in CTGD is controlled by the surface properties during pyrite formation rather than intrinsic properties of the crystal structure. Nevertheless, the crystal structure of pyrite needs to be sufficiently widened and modified to allow the adsorbed Au atoms to be incorporated and effectively sequestered from the fluid. The structural changes and a different bonding environment induced by the incorporation of As are, therefore, a key control of Au enrichment (*12*) and potentially define the solubility limit of Au^+1^ in pyrite. Although our newly constrained bulk partition data indirectly suggest that the Au enrichment into arsenian pyrite is rooted in processes happening at the fluid-mineral interface, our experimental setup is incapable of directly validating these processes. To answer the key questions of what happens at the interface (e.g., adsorption/desorption of different species, reduction/oxidation, and growth) and which physical-chemical reactions and properties (e.g., Au and As coordination in the pyrite structure) control the partitioning, direct spectroscopic techniques are needed, particularly, at elevated temperatures.

### Implications for the formation of giant ore deposits

The high partition coefficients of Au between fluid and pyrite (50 to 1800), which change as a function of the As concentration in pyrite, suggest that a new process can result in the efficient deposition of Au to form world class gold deposits. Enrichment of As in pyrite leads to high partition coefficients for Au between fluid and pyrite, producing pyrite that can effectively adsorb Au-HS complexes from the fluid onto the pyrite surface. Reactive Fe released by dissolution of Fe-bearing carbonates may destabilize the adsorbed Au-HS complexes by using the S of the complex to produce pyrite that is rich in structurally bound Au^+1^. As a consequence, precipitation of large amounts of fine-grained, or porous, pyrite with a large surface area is favorable for the formation of giant Au deposits. Partitioning provides an explanation for the occurrence of pyrite enriched in Au that forms before sulfidation and, therefore, has to be considered as an important ore-forming process in CTGDs. This study shows that trace element partitioning in hydrothermal environments, which is controlled by processes on the atomic scale, governs the effective enrichment and deposition of economically valuable elements, leading to the formation of giant ore deposits. Furthermore, we show how the incorporation of one trace element is coupled to another trace element, a fact that is rarely considered when trace elements are used as proxies for geological processes and sources.

## MATERIALS AND METHODS

### Partitioning experiments

Partitioning experiments were performed with in-house manufactured polytetrafluoroethylene beakers (~6 ml) that were closed by conical-shaped lids and placed in steel autoclaves. Autoclaves were placed in muffle furnaces at 200°C for varying run durations of 163 to 450 hours (table S1). The formation of pyrite was promoted by the interaction of natural siderite [Fe_0.6–0.9_(Mn,Mg)_0.1–0.4_CO_3_] from Bad Schlema, Germany, with aqueous fluid containing 0.05 *m* H_2_S. Thioacetamite (CH_3_CSNH_2_) was used as the sulfidation agent as it breaks down upon heating to form H_2_S. Experiments were conducted at fluid-buffered conditions and had high water-to-mineral ratios (by weight) of 1300 to 2250 (table S1). The molar ratio of H_2_S to Fe was always ~10 to ensure a constant H_2_S level in our experiments during pyrite formation. Therefore, the destabilization of sulfocomplexes due to pyrite formation is unlikely and did not influence partitioning. To control and fix the pH during experimental runs, most experiments were buffered to slightly acidic pH conditions between 4 and 6 by an acetate buffer solution (CH_3_COOH/CH_3_COONa) following Qian *et al*. (*37*). Trace amounts of arsenic and gold were added from an ICPMS standard solution [1000 μg/g in 2% HNO_3_ (As) and 1000 μg/g in 5% HCl (Au); Sigma-Aldrich] to achieve overall fluid concentrations ranging from 1 to 100 μg/g for As and 0.05 to 10 μg/g for Au (table S1). Thermodynamic modeling with the PHREEQC software package for experimental conditions and fluid compositions including pH buffer solutions indicated a maximum solubility limit of Au to be 0.6 μg/g (for pH 4 solution) and 5 μg/g (for pH 5 solution). Most experiments were undersaturated, with respect to metallic Au, and all added Au should be dissolved in the H_2_S-rich experimental fluid at 200°C. This can also be seen in the good correlation of Au concentration in pyrite with Au concentration in the fluid (Fig. 3, inset). If the fluid was largely supersaturated in metallic Au, then the Au concentration in the fluid would have been fixed at the solubility limit and the Au concentration of pyrite would have been the same regardless of the initial Au concentration of the experiment. As this not the case, we assumed the fluid to be undersaturated with respect to the native Au. The solubility modeling shows that Au-acetate and Au-Cl (sourced from thioacytamide, pH buffer, and standard solution) complexes are of very minor abundance, and almost all Au in solution is complexed by sulfides. Not all possible complexes (e.g., carbonic and ammonia) that might have formed during our experiments can be explored because of the lack of thermodynamic speciation data for Au. However, ligand exchange experiments for Au show that other complexes play a very minor role compared to sulfides (*38*). Given the high concentration of H_2_S (which forms immediately during heating) compared to low HCl concentrations from the standard solution, it is reasonable to assume that dissolved Au complexes equilibrate rather fast and transform from thermodynamically unstable Au-Cl to stable Au-HS complexes early within experimental runs.

In some experiments, beakers were flushed with Ar before closing to reduce fO_2_ of the experiment, which is induced by an air gap (table S1). Experiments were ended by taking the steel autoclaves out of the furnace and letting them cool slowly in air for about 1 hour before the beakers were removed from the autoclaves. Because of the relatively slow cooling, it cannot be ruled out that additional nanometer-sized mineral phases precipitated and have altered the fluid composition during cooling of the experiment. Nuggets of metallic Au form on the outside of replacement pyrite and pyrite seeds (fig. S1) in experiments done at high fluid Au concentrations of 5 to 10 μg/g; this potentially indicates Au precipitation during cooling. Because of the complex composition of experimental solids and fluids, additional nanometer-sized mineral phases (e.g., sulfides, carbonates, and oxides) might have formed during cooling and have changed the fluid composition. Immediately after ending the experiment, beakers were weighed to check for leakage and opened, and pH was measured. After separating fluid and solid run products, the fluids were acidified with 50-μl concentrated suprapure HNO_3,_ and solids were washed three times with deionized water and dried at 50°C. In many run solutions, a yellowish gel separated after acidifying, likely because of the reaction of the pH buffer with HNO_3_. It was not possible to redissolve this gel, which hindered the quantification of elements therein. Nevertheless, a small quantity of fluid was separated from the gel. This filtered experimental run solutions were measured with ICPMS, and transition metal concentrations were presented by Kusebauch *et al*. (*21*). Gold in the filtered run solutions ranged from 0.1 to 20 ng/g. Because of slow quenching of the experiments, the observed precipitation of Au nuggets, and the formation of the yellowish gel, these concentrations are highly fractionated and do not represent run fluid compositions at elevated temperatures. Although a mass balance for Au based on nugget occurrence, fluid, and pyrite chemistry was not possible, we interpreted our findings to imply a rather constant Au composition of the experimental fluid during pyrite formation [see section on Calculation of partition coefficients (*D* values) below]. Mass balance calculations based on averaged Au concentrations of the newly formed pyrite and the assumption that all dissolved Fe precipitated as pyrite show that, for most of the experiments, fluid concentrations have changed by less than 50%. Only few experiments (Sd2Py20, Sd2Py21, Sd2Py55, and Sd2Py56) might have experienced a larger change of up to ~80%. Nevertheless, these changes are comparably low compared to the overall compositional range of three orders of magnitude covered in this study, and only the last bit of pyrite would have seen a fully evolved fluid. In addition, the yellow color of the gel, which likely represents Au colloids, and Au nuggets formed on the outside of pyrite are suggesting high Au fluid concentrations at the end of the experimental run. One main mechanism to precipitate Au (either as nuggets or colloid) is the degassing of dissolved H_2_S from the quenched fluid after opening the beaker, oxidation, and acidification, as this will cause a subsequent supersaturation of Au, which is mainly complexed by sulfide.

### Mass spectroscopy (LA-ICPMS)

LA-ICPMS was carried out with an Analyte Excite 193 nm ArF Excimer-based LA System (Teledyne Photon Machines, Bozeman, MT, USA) coupled to a quadrupole ICPMS iCAP from Thermo Fisher Scientific. The LA system was equipped with a HelEx II 2-Volume ablation cell. Helium was used as a carrier gas for aerosol transport from the sample surface to the ICP and was mixed downstream with Ar as a make-up gas before entering the plasma. Operational parameters of the ICPMS instrument and LA unit were tuned for maximum sensitivity, low oxide formation based on the ^232^Th^16^O/^232^Th ratio, and low laser-induced elemental fractionation based on the ^238^U/^232^Th ratio with National Institute of Standards and Technology Standard Reference Material (NIST SRM) 610. For analysis, we measured the following isotopes: ^24^Mg, ^34^S, ^55^Mn, ^57^Fe, ^75^As, and ^197^Au. We used ^57^Fe as internal standard and the certified reference material MASS1 for calibration for all elements. Samples were ablated with spot sizes between 20 and 40 μm for 30 s, with a repetition rate of 10 Hz and an energy density of 2 to 3 J/cm^2^. The data were reduced with the commercial software Iolite (*39*) and the data reduction scheme X_trace_elemets_IS (*40*). Reproducibility of As concentrations was better than 7% on the basis of multiple measurements of standard material (i.e., NIST SRM 610 and MASS1).

### Calculation of partition coefficients (*D* values)

The experiments were designed in such a way to mimic conditions of natural CTGDs as close as possible (e.g., H_2_S, pH, fluid/rock ratio, and *T*) and meet experimental limitations and analytical requirements (e.g., safety and health regulations, experimental setup, amount of the newly formed pyrite, and the size of euhedral crystals). We performed our experiments in a largely fluid-buffered system with fluid/mineral ratios of >1000 to ensure a constant fluid composition and to precipitate sufficient amounts of pyrite for LA-ICPMS analysis. Nevertheless, the strong partitioning of As and Au observed in some experiments will lead to a sequestration of these two elements into a newly formed pyrite and, consequently, to a changing fluid composition throughout the experiment. Assuming constant *D* values for individual experiments, this compositional evolution of the fluid will be represented in the variation of pyrite composition within individual experiments. To account for the changing composition of the fluid and the resulting uncertainty for *D* value calculation, we applied three different approaches for the calculation of *D* values. In general, partition coefficients are expressed as *D* = *c*_(py)_/*c*_(fl)_, where *c*_(py)_ and *c*_(fl)_ are the concentrations of Au in coexisting pyrite and fluid, respectively. In the first approach, we calculated the *D* values (*D*_min_) for each experiment using the average pyrite Au concentrations measured by LA-ICPMS spots [*c*_(py)_] and assuming a constant Au concentration in the fluid, which is the starting concentration. In general, the *D* values calculated this way will give minimum values, as the real concentration of the fluid will be lower because of the sequestration into newly formed pyrite. In the second approach, we calculated the *D* values (*D*_max_) based on the average pyrite composition but a maximum evolved fluid composition having the lowest possible Au concentration. In this case, the Au concentration of the fluid was calculated from mass balance, assuming that all Fe from dissolution of siderite reprecipitated as pyrite. The amount of Au sequestered in this pyrite was calculated with the average pyrite Au concentration, which was subtracted from the starting composition of the fluid. By doing so, we got the highest possible evolved fluid composition, which was then used to calculate *D* values that, generally, will represent maximum values. The uncertainty for *D* of the first two methods is the SD of different LA-ICPMS measurements (table S2). In the third case, we modeled the fluid evolution and allocated the measured pyrite compositions to this evolution. In this way, we will get more realistic *D* values (*D*_opt_) that mostly falls between our minimum and maximum values. To account for compositional evolution of the fluid, we calculated the *D* value, following the assumption that pyrite having the highest measured Au concentration coexists with an unevolved fluid that also has the highest Au concentration (i.e., starting composition). We implemented a numerical mass balance model (see below) to calculate the expected compositional evolution of experimental fluid and coexisting pyrite based on experimental conditions. The mass balance model provides a theoretical composition of the last pyrite formed (table S2), which is in good agreement with the lowest actual measured pyrite composition of our experiment (table S2).

### Thermodynamic modeling

The solubility of Au was calculated with the PHREEQC software package with the implemented llnl.dat database for solids and major fluid species (*31*). In addition, stability constants (i.e., log *K*) for the major Au species [i.e., AuOH, AuCl_2_^−^, AuHS, and Au(HS)_2_^−^] were taken from Stefánsson and Seward (*41*, *42*) and were derived from experiments under conditions (i.e., H_2_S, *T*, and pressure) similar to CTGD. The solubility constants are in good agreement with more recent publications of Trigub *et al*. (*43*) and Pokrovski *et al*. (*25*, *26*). Gold solubility calculations were done under the following typical CTGD conditions: *T*, 200°C; pressure, 50 MPa (500 bar); 0.0001 to 0.1 *m* H_2_S; logfO_2_, ~−45; 2 *m* CO_2_; 1 *m* NaCl; neutral to slightly acidic pH (5.6 to 6); and siderite as the source of reactive iron (Table 1). An oxygen fugacity of logfO_2_ of ~−45 was used following considerations of Hofstra (*14*) (i.e., narrow stability field of cogenetic realgar observed in CTGD), predominance of CO_2_ over CH_4_, and calculated fO_2_ of siderite and calcite dissolution. The chosen fO_2_ value falls between the hematite-magnetite buffer (logfO_2_ = −43) and the pyrite-pyrrhotite-magnetite buffer (logfO_2_ = −47.3). The pH and fO_2_ in all calculations were buffered by calcite dissolution and dissolved carbonate-CO_2_ equilibria. The effect of different pH and fO_2_ on Au solubility is discussed in the Supplementary Materials. In all calculations, the complex Au(HS)_2_^−^ is by far the most important complex under these conditions (*25*, *26*, *41*).

### Numerical mass balance model

The compositional evolution of fluid and pyrite during CTGD formation (and within our experimental runs) was numerically modeled to investigate the influence of the two possible Au-sequestering processes: partitioning during pyrite formation and supersaturation of Au caused by sulfidation. As boundary conditions for the model, we assumed a closed system (similar to batch experiments), no re-equilibration of already precipitated pyrite, the amount of pyrite [*m*_py(i)_] formed between each step as constant, the pyrite as the only Fe and S sink that was always supersaturated, and all reactive Fe were completely reacted. From the initially defined amount of reactive Fe (*m*_Fe_) (i.e., Fe that is either bound to carbonates and oxides or dissolved in an additional fluid but not bound to sulfides or silicates) and H_2_S molar concentration (*c*_H2S_) of the fluid, the amount of fluid (m_H2O_) that is needed to completely react the reactive Fe to form pyrite can be calculated. Initial conditions and composition of the fluid for our model are given in Table 1. All discussed variables and their range are based on observations in natural CTGDs (Table 1). The total mass of Au (*m*_Au-total_) in the system is given by the total amount of fluid and the initial fluid Au concentration [*c*_Au-Fl(0)_] as

$$m_{\text{Au}-\text{total}}=m_{\text{Fl}}\times c_{\text{Au}-\text{Fl}(0)}$$ (1)

During each iteration of the numerical model, a constant fraction of Fe and S was used to form stoichiometric pyrite. The mass of Au [*m*_Au(i)_] contained in this newly formed pyrite is calculated with our newly constrained partition coefficients (*D*_Au_ = 1000) (Fig. 3) as

$$m_{\text{Au}-\text{Py}(i)}=c_{\text{Au}-\text{Fl}(i-1)}\times D_{\text{Au}}\times m_{\text{py}(i)}$$ (2)

For the next step, the amount of Au in the fluid is reduced by the amount of Au fixed in the pyrite produced in the previous step using

$$m_{\text{Au}-\text{Fl}(i)}=m_{\text{Au}-\text{Fl}(i-1)}-m_{\text{Au}-\text{Py}(i)}$$ (3)

The numerical model continues until the H_2_S concentration of pyrite supersaturation (~0.0001 *m* H_2_S at the given logfO_2_ of ~−45) is reached and until almost all reactive Fe and S are consumed.

The onset of sulfidation in our model is equal to the point at which the solubility limit of Au is reached, taking into account the ongoing depletion of Au and H_2_S in the fluid by partitioning. When the solubility limit is reached, sulfidation becomes the governing process and Au concentration follows the solubility limit (Fig. 4). The Au concentration in pyrite is modeled, following equilibrium partitioning before the onset of sulfidation or assuming that supersaturated Au precipitates together with pyrite.

## Supplementary Material

http://advances.sciencemag.org/cgi/content/full/5/5/eaav5891/DC1

Download PDF

## SUPPLEMENTARY MATERIALS

Supplementary material for this article is available at http://advances.sciencemag.org/cgi/content/full/5/5/eaav5891/DC1 Supplementary Text Fig. S1. Au nuggets formation on the outside of pyrite. Fig. S2. Time resolved LA-ICPMS spectra. Fig. S3. Dependency of the modeled Au evolution on *D* values and initial Au concentration. Fig. S4. Dependency of the modeled Au evolution depending on different Au solubilities calculated for different fO_2_ and pH and constant boundary conditions. Table S1. Experimental conditions. Table S2. As and Au concentrations (in μg/g) of experimental pyrite measured by LA-ICPMS and calculated *D* values. Table S3. Sources of thermodynamic data for species and minerals used in this study. References (*44*–*46*)
