# Supplementary material for: Coupled partitioning of Au and As into pyrite controls formation of giant Au deposits
Source: Sci Adv. 2019 May 1;5(5):eaav5891. doi: 10.1126/sciadv.aav5891 (PMC6494509; doi:10.1126/sciadv.aav5891)
Supplement: http://advances.sciencemag.org/cgi/content/full/5/5/eaav5891/DC1 [file supp_5_5_eaav5891__index.html]

Science Advances | Science Advances

## Supplementary Materials

**This PDF file includes:**

- Supplementary Text
- Fig. S1. Au nuggets formation on the outside of pyrite.
- Fig. S2. Time resolved LA-ICPMS spectra.
- Fig. S3. Dependency of the modeled Au evolution on *D* values and initial Au concentration.
- Fig. S4. Dependency of the modeled Au evolution depending on different Au solubilities calculated for different fO2 and pH and constant boundary conditions.
- Table S1. Experimental conditions.
- Table S2. As and Au concentrations (in μg/g) of experimental pyrite measured by LA-ICPMS and calculated *D* values.
- Table S3. Sources of thermodynamic data for species and minerals used in this study.
- References (*44*–*46*)

Download PDF

**Files in this Data Supplement:**

- Adobe PDF - aav5891\_SM.pdf
