# Supplementary material for: Coupled partitioning of Au and As into pyrite controls formation of giant Au deposits
Source: Sci Adv. 2019 May 1;5(5):eaav5891. doi: 10.1126/sciadv.aav5891 (PMC6494509; doi:10.1126/sciadv.aav5891)
Supplement: Download PDF [file aav5891_SM.pdf]

Science Advances
AAAS

advances.sciencemag.org/cgi/content/full/5/5/eaav5891/DC1

Supplementary Materials for

Coupled partitioning of Au and As into pyrite controls formation of giant Au deposits

C. Kusebauch*, S. A. Gleeson, M. Oelze

*Corresponding author. Email: c.kusebauch@gfz-potsdam.de

Published 1 May 2019, *Sci. Adv.* **5**, eaav5891 (2019)
DOI: 10.1126/sciadv.aav5891

This PDF file includes:

Supplementary Text
Fig. S1. Au nuggets formation on the outside of pyrite.
Fig. S2. Time resolved LA-ICPMS spectra.
Fig. S3. Dependency of the modeled Au evolution on *D* values and initial Au concentration.
Fig. S4. Dependency of the modeled Au evolution depending on different Au solubilities calculated for different fO_2 and pH and constant boundary conditions.
Table S1. Experimental conditions.
Table S2. As and Au concentrations (in μg/g) of experimental pyrite measured by LA-ICPMS and calculated *D* values.
Table S3. Sources of thermodynamic data for species and minerals used in this study.
References (*44–46*)

Supplementary Text

Newly formed pyrite from replacement of siderite and LA-ICPMS spot selection

Hydrothermal pyrite forms via fluid mediated coupled dissolution-reprecipitation of siderite (*21*). Replacement of siderite can be described as pseudomorphic as the former shape is preserved either by porous pyrite or clusters of euhedral, newly formed, pyrite (Fig. 1 and S1). Spots for LA-ICPMS were set on areas that have a high density of euhedral pyrite as it was found to represent pyrite that had equilibrated with the experimental fluid. In contrast, porous pyrite formed at an early stage as an immediate product of siderite replacement (*21*). Run time dependent experiments suggest that euhedral pyrite forms from reprecipitation of porous pyrite and its abundance increases with run duration (*21*). Duration and pH for partitioning experiments were optimized to produce euhedral pyrite. As well as pyrite, minor amounts of pyrrhotite are found in most experiments but EMPA measurements confirm that it does not contain significant concentrations of Au and As.

Pyrite formed in experiments with the highest Au concentrations contains visible Au nuggets (fig. S1) in addition to dissolved Au in the pyrite structure. These findings are in agreement with a postulated solubility limit of Au^{+1} in arsenian pyrite (*10, 11*) of $Au=0.02As+4x10^{-5}$ (Fig. 2). Gold nuggets were also detected during LA-ICPMS measurements and appear as spikes in the time resolved ablation spectra (fig. S2). These spikes were excluded during data processing used for calculation of concentrations and, therefore, the presented Au concentrations should represent only dissolved Au^{+1}. Nevertheless, pyrite compositions plot above the empirically defined solubility limit for some high Au experiments (Fig. 2). This pyrite might either contain nano-nuggets of native Au that cannot be resolved by LA-ICPMS (<100nm, which is roughly the ablation depth of one laser pulse) or this pyrite deviates from the empirically defined solubility limit. Deviation from the solubility limit was observed in previous studies and seems to be controlled by kinetics (*8, 44, 45*), which might also play a role in our experiments.

Although, Au nano-nuggets might occur, they do not influence our D value calculations as these are purely empirical and only express the relationship between fluid composition and pyrite composition in experiment and in nature. Additionally, the majority of experiments gave pyrite with Au concentrations below the solubility limit and, therefore, Au in pyrite should be only structurally bound.

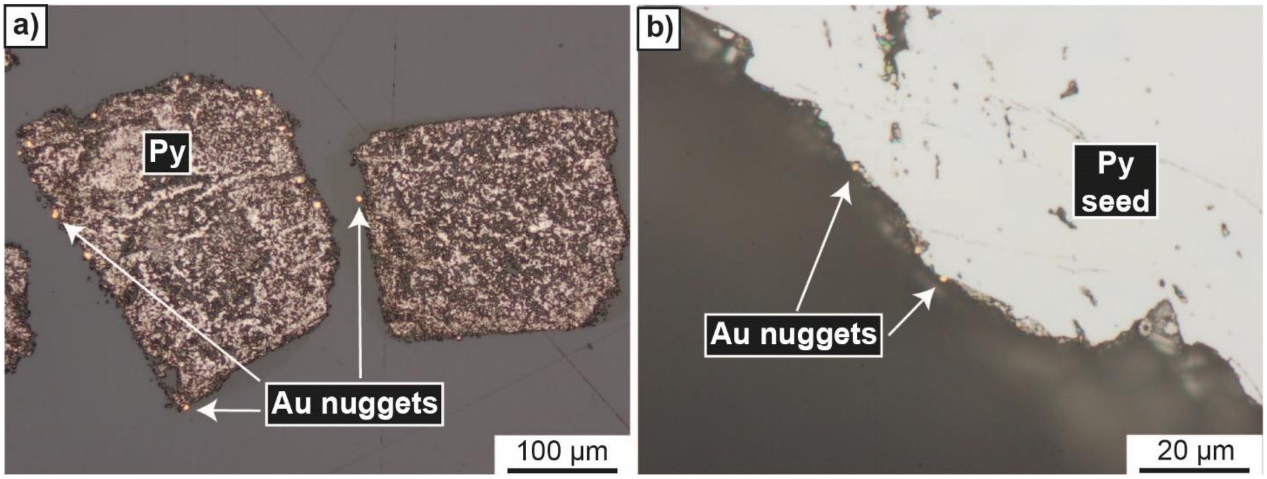

Fig. S1. Au nuggets formation on the outside of pyrite. Nuggets of native Au forming on the outside of **a**) replacement pyrite during experiments with high Au concentrations (Sd2Py47, Au: 5ppm); **b**) on pyrite seeds (Sd2Py26, Au:10ppm).

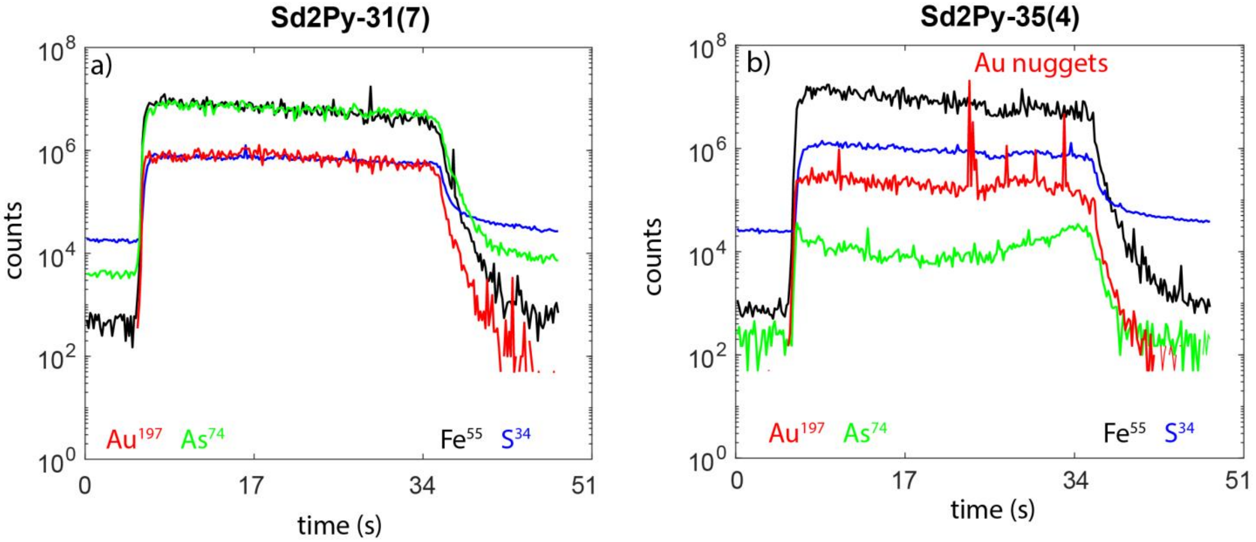

Fig. S2. Time resolved LA-ICPMS spectra. Examples of time resolved LA-ICPMS spectra for nugget free (**a**: Sd2Py-31) and nugget containing (**b**: Sd2Py-35) pyrite.

Comparison of modelled features with natural observations

Ore grade and fluid-rock ratio

Apart from the compositional evolution of fluid and pyrite as a function of pyritization, our mass balance model also allows for calculation of total ore grades and fluid rock ratios. Critical for these calculations are the given assumptions that the amount of reactive Fe in our system controls the amount of H_2S of the model and, therefore, the overall amount of fluid and the amount of Au. Given the conditions for our main model presented in Fig. 4 and a complete pyritization process, modelled whole rock ore grades increase from 14 to 143 g/T with decreasing H_2S concentration of the fluid from 0.1 molar to 0.01 molar. At the same time, the fluid-to-rock ratio (f/r) increases from 7 to 72 with decreasing H_2S concentration of the fluid.

In general, ore grades of CTGD from Nevada are in the range of <1 up to 29 g/T (*10, 11*) and are in agreement with our calculations for H_2S enriched fluids having > 0.05 molar H_2S. In contrast, fluid-to-rock ratios from the literature disagree with our calculations. In Cline et al. (2005)(*2*) the f/r ratio is calculated to be 525-785 based on the dissolution of calcite and dolomite. Hofstra et al. (1991) (*14*) calculate f/r ratio on the basis of mass balance (assuming sulfidation as the only Au depositing process) and thermodynamic modeling to be in the order of 50-200 for host rocks containing 1wt.% reactive Fe. In both cases, f/r ratios differ from our calculations for different reasons. In the case of Cline et al. (2005), carbonate dissolution is not necessarily linked to ore formation and is likely a later phenomenon due to alteration, a fact that the authors state themselves. The f/r ratios based on decarbonisation are, therefore, not representing the amount of ore fluids. In the case of Hofstra et al. (1991), the assumption of sulfidation being the only Au depositing process shifts f/r ratios to higher values. As we show with our data, deposition of Au occurs already at Au undersaturated conditions and, therefore, low fluid amounts are calculated by our model.

Compositional features of CTGD pyrite

Gold in ore stage pyrite of CTGD is distributed heterogeneously (*15*) and although compositions range from few µg/g up a few thousand µg/g within one deposit, the majority of data falls within a range from tens to hundreds of µg/g (Fig. 2). The process forming CTGD must be able to produce the observed compositional variability in agreement with the abundance of pyrite. If

sulfidation is the governing ore forming process throughout the whole fluid-rock interaction sequence (i.e., supersaturation of Au at early stages), newly formed pyrite will have homogenously high Au concentrations during a large part of the pyritization process. Only at the end when there is little H_2S is left (i.e., at high degrees of pyritization) will pyrite have low Au concentrations. In contrast, partitioning will deplete Au in the fluid from the beginning on resulting in pyrite compositions that cover the whole range of Au concentrations observed in CTGD pyrite (the blue line in Fig. 4).

Apart from looking at the compositional variance of pyrite on a deposit scale, compositional changes in the fluid will also have an impact on the spatial composition of pyrite grains that continuously grow from that fluid. Recently published nanoSIMS data of pyrite from Lannigou Carlin style deposits show the highest Au concentrations are in earliest ore stage pyrite that forms an overgrowth on preexisting barren pyrite (*28*). Gold concentrations decrease with increasing distance from the interface of the overgrowth and track a compositional evolution of the fluid. If the Au precipitated as a result of supersaturation induced by pyrite precipitation, Au concentrations will form a plateau (similar to the red line in fig. S4) rather than a local maximum as the H_2S concentration of the fluid will be sufficiently high considering how much pyrite is formed after the Au maximum. Partitioning on the other site will produce strongly decreasing fluid compositions that leaves its imprint in the pyrite composition formed in local equilibrium with the fluid.

Using different boundary conditions for mass balance model for CTGD deposits

To study the influence of different boundary conditions on our mass balance model we changed the amounts of reactive iron, initial Au, D values and Au solubility (as a function of fO_2 and pH) independently. The first 3 variables (i.e., reactive Fe, initial Au, D values) were modelled using a fixed $logfO_2$ (-43) and fixed pH (5.3). The influence of a changed solubility limit is investigated using a fixed H_2S (0.05 molal), fixed Au concentration 2 µg/g, fixed reactive Fe (2wt%) and a D value of 1000.

Dependency of the model on different amounts of reactive iron

As the amount of reactive Fe is coupled to the amount of fluid having a given H_2S concentration, changing reactive Fe will change the fluid/rock ratio and, therefore the ore grade. The ore grade will be reduced to 3.6 g/t for 0.1 molal H_2S and 36 g/t for 0.01 molal H_2S, respectively. All other features will be preserved.

Dependency of the model on different D values

Partition coefficients for Au depend on As composition of newly formed pyrite (Fig. 3a). For the presented modeling in Fig. 4 the D value was chosen to be 1000 as CTGD pyrite has characteristically high As concentrations of over 3 wt.%. Using a lower D value of 100, which is found for low As pyrite, leads to changes in the modelling results. Pyrite originating from a fluid that has the same Au concentrations as in the main model (2µg/g, dotted lines in fig. S3) will have Au concentrations 10 times lower due to the lower D value. Therefore, the scavenging of Au from the fluid becomes ineffective and partitioning will not sequester much Au before the onset of sulfidation (fig. S3a). Natural pyrite formed under this condition (i.e., low As) will contain large zones with low Au contents that are grown in undersaturated conditions but also zones having high concentrations of native Au when supersaturation is reached.

Dependency of the model on different initial Au concentrations

Changing Au concentrations will affect the Au grades, but more importantly the onset of sulfidation as the solubility limit will be reached at different degrees of pyritization (fig. S3b). In the main case (Fig. 4), the chosen initial Au concentration (2µg/g) is relatively high to meet average compositions of (the only) published fluid inclusion and pyrite data for Guizhou Carlin-style deposits (*9*). Average Au concentrations in CTGD pyrite from Nevada are generally one to

two orders of magnitude lower and only have rarely concentrations above 1000µg/g (*10, 11, 16*). The lower pyrite Au concentrations indicate one to two orders of magnitude lower fluid Au concentrations (compared to the Guizhou Carlin-style deposits) that are generally below detection limits of analytical techniques. Nevertheless, inferred initial fluid concentrations of 0.5 µg/g Au for Nevada CTGD will produce early pyrite having 500µg/g Au and due to the elongated decrease of Au concentration of the fluid that is caused by partitioning, later pyrite will span the whole range of observed Au concentrations of pyrite from Nevada CTGD (fig. S3b).

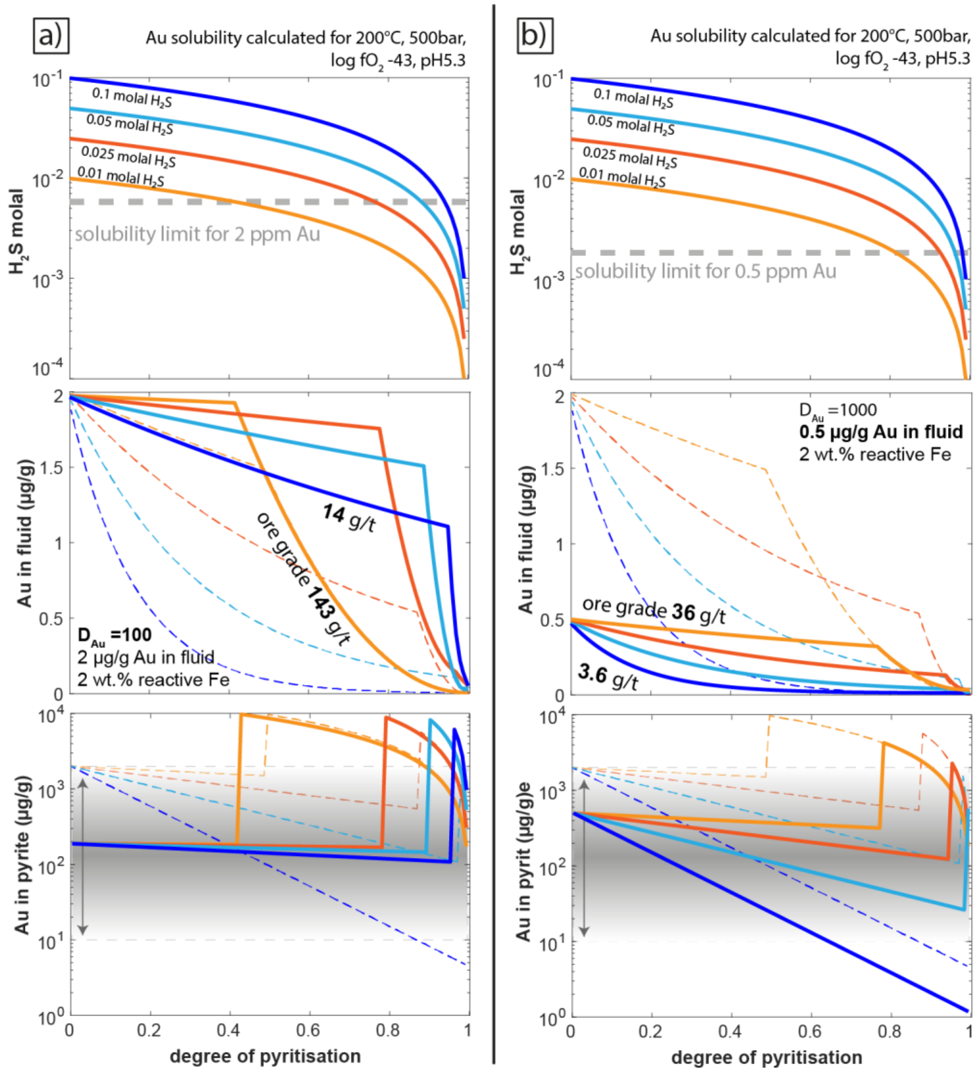

Fig. S3. Dependency of the modeled Au evolution on *D* values and initial Au concentration. Dependency of the modeled Au evolution (solid lines) on: **a**) the used partition coefficients (D= 100) for Au between fluid and pyrite, lower D values will change the onset of sulfidation and lower the Au enrichment before sulfidation, characteristic ore systems (i.e., low As) will show Au nuggets rather than incorporated Au; **b**) the initial Au concentration in the fluid; lower Au concentrations in the fluid of 0.5 µg/g (realistic for CTGD) will lead to lower ore grades and a shift of sulfidation to higher degrees of pyritisation, consequently, scavenging of Au by partitioning will become more important in these CTGD systems; for comparison: dashed lines from the model using assumptions presented in the main text (i.e., D value: 1000, $Au_{(Fl)}$: 2µg/g, reactive Fe: 2wt%).

Dependency of the model on different Au solubility as a function of fO_2 and pH

One critical parameter during the modelling is the Au solubility limit, which strongly influences the onset of sulfidation. Thermodynamic modelling of the solubility limit was done using the PHREEQC software package combined with experimentally derived solubility constants for Au complexes from Stefansson and Seward (2003, 2004) (*41*, *12*). These data are in agreement with more recent studies by Pokrovski et al. (2009) (*26*) and Tribut et al. (2014) (*43*) and are used for the calculation of thermodynamic properties of Au-HS complexes (Pokrovski et al., 2014) (*25*). We chose solubility constants from Stefansson and Seward (2003, 2004) as they were derived from experiments done under pressure, temperature and H_2S conditions representative of initial CTGD fluids. The solubility constants used in our model are 3-4 orders of magnitude higher compared to these previously used (*46*) in thermodynamic models of CTGD sulfidation (*2*, *7*) and will give much higher solubility limits (see Pokrovski et al., 2009 for detailed discussion of solubility constants). Additional, to the uncertainties introduced by different thermodynamic data, the choice of fO_2 and pH is critical for Au solubility calculation in general and in our model in particular. A general increase of the solubility limit of Au will lead to the preferential sequestration of Au by partitioning as more pyrite will precipitate before the Au saturation is reached. Contrastingly, a lower solubility limit will prefer sulfidation as the ore depositing process is the destruction of Au-HS complexes and will lead to an oversaturation in respect to Au metal. In CTGD systems, Au solubility has its maximum in the stability field of pyrite at a pH between 6.5-7 and a fO_2 close to the magnetite-hematite buffer (see Hofstra and Cline, 2000). Additionally to the loss of S during pyritisation (Fig. 4, S3, S4), a decreasing pH or oxygen fugacity can lead to a super-saturation of Au. For our main model, the pH (~5.6) and fO_2 ($\log fO_2$ ~-45) were constant and defined by solid buffer during pyritisation. These assumptions are justified by the constant dissolution of carbonates (i.e., siderite and calcite) and resulting establishment of a dissolved carbonate-CO_2 pH buffer system at ~5.6-6. Similarly, the $\log fO_2$ is calculated to be ~-45 by the dissolution of carbonates The $\log fO_2$ falls between the hematite-magnetite (HM) and pyrrhotite-pyrite-magnetite (PPM) buffer and is in agreement with previous studies (*9*, *10*, *20*). To investigate the influence of a changing pH and fO_2 on our mass balance model, we calculated Au solubility limits for different pH values (pH 4, pH 5) and $\log fO_2$ (-43=HM and -47 = PPM). For these models following conditions were used: H_2S = 0.05 molal; Au_{fl} = 2 µg/g; reactive Fe = 2 wt%; D_{Au}=1000. In these cases the fluids were not buffered by carbonate equilibria but fixed to the given pH and fO_2. In general, the shift of the solubility limit

will change the onset of sulfidation (fig. S4). In all cases (except for pH 4) the scavenging of Au by partitioning will be the major process of Au deposition and only at the very end of pyritization of the wall rock, supersaturation will be achieved. In the case of pH 4, the initial fluid is already supersaturated and fluid as well as pyrite Au concentrations follow the solubility limit curve. In this case, sulfidation is the governing process since no pyrite is precipitated at undersaturated condition.

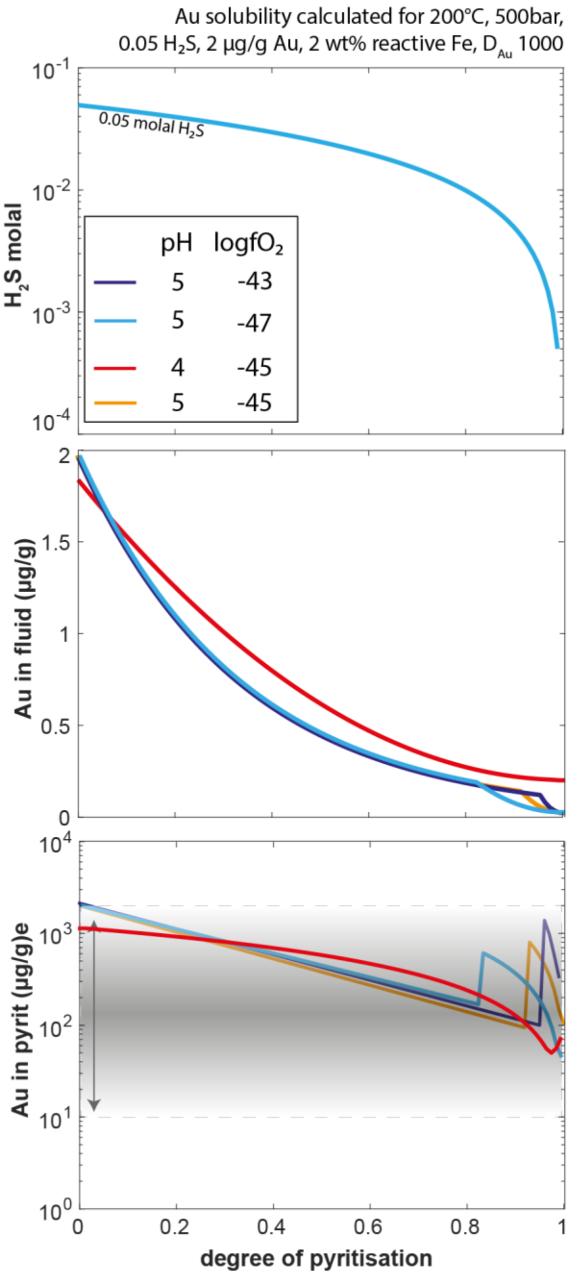

Fig. S4. Dependency of the modeled Au evolution depending on different Au solubilities calculated for different fO_2 and pH and constant boundary conditions.

Table S1. Experimental conditions.

Sample	T (°C)	t(h)	sol. As (μg/g)	sol. Au (μg/g)	pH buffer	pH calc @200°C[§]	$pH_{25°C}$ after exp	w/r	molal H2S in fluid	media above fluid	assemblage
Sd2Py24	200	163	0	0.1	unbuffered		5.9	1691	0.056	Ar	Py
Sd2Py25	200	163	0	1	unbuffered		7.3	1761	0.050	Ar	Py
Sd2Py26	200	163	0	10	unbuffered		5.2	1841	0.050	Ar	Py
Sd2Py30	200	163	100	0.1	unbuffered		9.4	1557	0.047	Ar	Py, +-Po
Sd2Py31	200	163	100	1	unbuffered		9.6	1841	0.047	Ar	Py, +-Po
Sd2Py32	200	163	100	10	unbuffered		9.1	1300	0.048	Ar	Py, +-Po
Sd2Py33	200	450	1	0.05	pH4*	4.7	4.3	1758	0.056	Ar	Py, +-Po
Sd2Py34	200	450	1	0.5	pH4*	4.7	4.3	2240	0.052	Ar	Py, +-Po
Sd2Py35	200	450	1	5	pH4*	4.7	4.2	1610	0.053	Ar	Py, +-Po
Sd2Py36	200	450	5	0.05	pH4*	4.7	4.4	2235	0.051	Ar	Py, +-Po
Sd2Py37	200	450	5	0.5	pH4*	4.7	4.4	2116	0.053	Ar	Py, +-Po
Sd2Py38	200	450	5	5	pH4*	4.7	4.3	1916	0.051	Ar	Py, +-Po
Sd2Py45	200	168	50	0.05	pH5*	5.8	5.4	2124	0.051	air	Py, +-Po
Sd2Py46	200	168	50	0.5	pH5*	5.8	5.4	1683	0.049	air	Py, +-Po
Sd2Py47	200	168	50	5	pH5*	5.8	5.3	1609	0.050	air	Py, +-Po
Sd2Py48	200	168	100	0.1	pH5*	5.8	8.1	1561	0.053	air	Py, +-Po
Sd2Py49	200	168	100	1	pH5*	5.8	8.8	2028	0.049	air	Py, +-Po
Sd2Py50	200	168	100	10	pH5*	5.8	7.9	1283	0.062	air	Py, +-Po
Sd2Py51	200	332	10	0.05	pH5*	5.8	5.1	1642	0.051	air	Py, +-Po
Sd2Py52	200	332	10	0.1	pH5*	5.8	5.2	1633	0.051	air	Py, +-Po
Sd2Py53	200	332	10	0.5	pH5*	5.8	5.2	1515	0.053	air	Py, +-Po
Sd2Py54	200	332	20	0.05	pH5*	5.8	5.2	1560	0.055	air	Py, +-Po
Sd2Py55	200	332	20	0.1	pH5*	5.8	5.2	1552	0.054	air	Py, +-Po
Sd2Py56	200	332	20	0.5	pH5*	5.8	5.2	1500	0.053	air	Py, +-Po

* pH buffer CH2COOH/CH3OONa: 0.1656M /0.0343M (for pH 4); 0.06/0.14 (for pH5)

§ calculated using PHREEQC and implemented llnl.dat database

Compositions of experimentally formed pyrite

Table S2. As and Au concentrations (in µg/g) of experimental pyrite measured by LA-ICPMS and calculated *D* values.

Exp		sol. As (µg/g)	sol. Au (µg/g)	As in py (µg/g)		s.d.	D_{As}	Au in py (µg/g)		s.d.	D_{Au-min}			D_{Au-max}			D_{Au-opt}	Au_{min} LA-ICPMS (µg/g)	Au_{min} model (µg/g)
Sd2Py	24	0	0.1					9.5	±	5.1	95	±	51	100	±	53	151	3	14
Sd2Py	25	0	1					130.0	±	41.7	130	±	42	141	±	43	169	77	153
Sd2Py	26	0	10					1928.2	±	777.1	193	±	78	216	±	81	330	1009	2746
Sd2Py	30	100	0.1	71461	±	24902	1067	120.2	±	20.4	1202	±	204	5976	±	236	1466	95	56
Sd2Py	31	100	1	50450	±	18075	776	1231.5	±	103.2	1232	±	103	4005	±	110	1341	1070	634
Sd2Py	32	100	10	42466	±	5709	558	6839.0	±	467.6	684	±	47	1501	±	49	738	5990	4118
Sd2Py	33	1	0.05	1282	±	833	2116	2.9	±	0.3	58	±	6	60	±	6	65	3	3
Sd2Py	34	1	0.5	1545	±	463	2200	58.2	±	16.8	116	±	34	123	±	34	142	34	67
Sd2Py	35	1	5	224	±	141	359	298.3	±	110.9	60	±	22	62	±	22	85	219	403
Sd2Py	36	5	0.05	4420	±	2147	1594	3.5	±	0.9	71	±	18	73	±	18	95	2	5
Sd2Py	37	5	0.5	3994	±	2375	1196	15.3	±	8.8	31	±	18	31	±	18	51	9	25
Sd2Py	38	5	5	4515	±	2490	1612	529.4	±	113.0	106	±	23	112	±	23	137	337	637
Sd2Py	45	50	0.05	38700	±	15771	1147	26.5	±	3.6	529	±	71	714	±	74	624	23	23
Sd2Py	46	50	0.5	32086	±	6641	773	251.3	±	85.2	503	±	170	728	±	190	822	158	249
Sd2Py	47	50	5	34485	±	4527	765	4400.0	±	901.5	880	±	180	2028	±	204	1234	3870	2803
Sd2Py	48	100	0.1	30065	±	2923	332	84.5	±	15.8	845	±	158	1919	±	177	1016	56	52
Sd2Py	49	100	1	68688	±	14609	927	1019.3	±	138.2	1019	±	138	2124	±	149	1209	847	655
Sd2Py	50	100	10	31703	±	4106	376	5656.7	±	381.1	566	±	38	1041	±	39	607	4820	3733
Sd2Py	51	10	0.05	16760	±	2532	1990	31.9	±	5.9	639	±	117	1069	±	127	822	24	25
Sd2Py	52	10	0.1	12336	±	4175	2060	35.2	±	6.8	352	±	68	453	±	72	489	27	36
Sd2Py	53	10	0.5	15966	±	6192	2660	304.0	±	87.5	608	±	175	1040	±	199	964	228	251
Sd2Py	54	20	0.05	21137	±	3729	1270	22.3	±	5.2	446	±	103	633	±	111	648	17	21
Sd2Py	55	20	0.1	26183	±	9615	1885	114.0	±	52.3	1140	±	523	4758	±	803	1804	64	54
Sd2Py	56	20	0.5	25213	±	3304	1450	598.5	±	110.9	1197	±	222	6878	±	262	1526	449	268

Data used for thermodynamic calculations

Table S3. Sources of thermodynamic data for species and minerals used in this study.

Species and phases	Data source
**Aqueous species**	
H_2O, H^+, OH-, H_2S, HS-, $H_2S_2O_3$, $HS_2O_3^-$, $S_2O_3^{2-}$, HSO_3^-, SO_3^{2-}, HSO_4^-, SO_4^{2-}, $H_2S_2O_4^0$, $HS_2O_4^-$, $S_2O_4^{2-}$, $S_2O_5^{2-}$, CH_4, CO_2, H_2CO_3, HCO_3^-, Cl^-, Na^+, $NaCl^0$, $NaHCO_3$, $NaCO_3^-$ $NaOH^0$, HCl^0, SO_2^0, H_2^0, O_2^0, S^{2-}, S_2^{2-}, S_3^{2-}, S_4^{2-}, S_5^{2-}, NH_4^+, NH_3, N_2,	llnl.dat database of Parkhurst et al. (2013)
Fe^{2+}, $FeHCO_3^+$, $FeCO_3$, $FeOH^+$, $Fe(OH)_2$, $Fe(OH)_3^-$, $Fe(OH)_4^{2-}$, $FeSO_4$, $FeSO_4^+$, $FeSO_4^{2-}$, $FeCl_4^{2-}$, $FeCl^+$, $FeCl_2$, Ca_2^+, $CaCl^+$, CaCl2, $CaHCO_3^+$, $CaCO_3$, $CaSO_4$, $CaOH^+$, Acetate, AuAcetate, FeAcetate, $Acetate^-$, HAcetate, NaAcetate, NH_4Acetate, $NH_4(Acetate)_2^-$	llnl.dat database of Parkhurst et al. (2013)
Au^{+*}, $AuOH^{0§}$, $Au(Cl)_2^{-§}$, $AuHS^{0*}$, $Au(HS)_2^{-*}$	* Stefansson and Seward (2004), § Stefansson and Seward (2003)
**Solids**	
Siderite, Calcite, Pyrite, Pyrrhotite, Hematite, Magnetite, Gold	llnl.dat database of Parkhurst et al. (2013)
